# Region-aware bridge modeling enables interpretable mesoscale representation of spatial transcriptomic tissue sections

**DOI:** 10.1093/bioadv/vbag176

**Published:** 2026-06-23

**Authors:** Seung-Hwan Kim

**Affiliations:** Department of Biology, Fisher College, Boston, MA, United States; Department of Pediatric Oncology, Dana-Farber Cancer Institute, Boston, MA, United States

## Abstract

**Motivation:**

Spatial transcriptomics maps tissue architecture at high resolution, but spot-level maps are difficult to compare across sections, whereas whole-section averages obscure regional organization. We developed region-aware bridge modeling to aggregate interpretable biological program features into compact mesoscale summaries.

**Results:**

Using public colorectal cancer and breast cancer 10x Genomics high-definition Visium sections, we constructed epithelial-like, fibroblast, smooth or myoepithelial, and extracellular matrix bridge features from cell-state indicators, curated gene-program scores, and quality-control summaries. Median-quadrant aggregation produced an eight-region design matrix. Within-section validation showed non-random mesoscale heterogeneity: region-label shuffling reduced the overall mean absolute pairwise regional difference from 0.298 to 0.0020 in colorectal cancer and from 0.328 to 0.0024 in breast cancer, with empirical upper-tail probability values of 0.0002 for both analyses across 5000 permutations. Shifted and rotated partitions changed the overall heterogeneity metric by less than 10%. Exploratory ridge modeling identified fibroblast as the only non-target predictor in the extracellular matrix model, and Bayesian sensitivity analysis supported a positive fibroblast-extracellular matrix association. Supplementary lung, prostate, and ovarian cancer sections supported workflow applicability.

**Availability and implementation:**

Code and processed outputs are available through GitHub and Zenodo under 10.5281/zenodo.19801620.

## 1 Introduction

Spatial transcriptomics has transformed the study of tumor ecosystems by enabling gene expression to be measured in tissue context within intact specimens (Ståhl *et al*. 2016, Rao *et al*. 2021, Lewis *et al*. 2021, Elhanani *et al*. 2023, Walsh and Quail 2023, Gong *et al*. 2024). Rather than treating tumors as spatially homogeneous mixtures, these technologies enable epithelial, stromal, immune, vascular, and extracellular matrix programs to be localized across tissue space and related to tumor organization, progression, and therapeutic response (Rao *et al*. 2021, Feng *et al*. 2024, Aung *et al*. 2025, Liu *et al*. 2025). This opportunity is especially important for solid tumors, in which regional microenvironmental structure can carry information that is obscured by whole-section or cohort-level averages (Jing *et al*. 2025, Sharma *et al*. 2025).

A central challenge is how to represent spatial complexity at an interpretable intermediate scale. Spot-level or bin-level analyses preserve high spatial resolution, but they can be difficult to summarize across sections, compare across samples, or incorporate into downstream statistical models (Fang *et al.* 2025, Ke *et al*. 2025, McKenzie *et al*. 2026). At the opposite extreme, whole-section averages are compact but may obscure biologically meaningful regional structure. Recent computational frameworks have addressed related problems, including multiscale spatial analysis ecosystems, spatially aware dimension reduction, comparative multi-sample spatial analysis, spatial relationship modeling, and graph-based tumor heterogeneity scoring (Martinelli and Rapsomaniki 2022, Tanevski *et al*. 2022, Zhong *et al*. 2024, Chen e*t al*. 2025, Fang *et al*. 2025, Tang *et al*. 2026). However, there remains a need for lightweight and reproducible representations that aggregate local biological programs into compact region-level summaries while retaining interpretable spatial organization.

Tumor microenvironments are shaped by interacting epithelial, stromal, contractile, and matrix-associated programs rather than by isolated cell states alone (Lendahl *et al*. 2022, Liu *et al*. 2025, Pentimalli *et al*. 2025). Fibroblast-rich and ECM-rich regions can mark structural microenvironmental niches, whereas epithelial and myoepithelial or smooth-muscle-associated programs can define distinct tissue architectures and tumor boundaries (Feng *et al*. 2024, Gray *et al*. 2025). A region-aware representation that links these axes into a shared mesoscale summary could therefore provide a useful bridge between high-resolution spatial maps and compact statistical models.

Here, we develop a region-aware bridge modeling framework for constructing interpretable mesoscale representations of spatial transcriptomic tissue sections. Using one public colorectal cancer (CRC) Visium HD section and one public breast cancer (BC) Visium HD section as a proof-of-concept, we construct a transparent spot-level bridge-target representation from coarse cell-state indicators, curated gene-program scores, and quality-control summaries. We focus on four core bridge features—epithelial-like, fibroblast, smooth/myoepithelial, and extracellular matrix (ECM)—and aggregate these features within median-based spatial quadrants to form a compact CRC–BC region-aware design matrix. Because the primary analysis contains one section per tumor type and eight region-level observations, all validation is interpreted as within-section validation, and ridge and Bayesian models are interpreted as exploratory association and sensitivity analyses rather than confirmatory predictive or causal models.

We show that median-quadrant bridge summaries preserve non-random mesoscale heterogeneity that is obscured by whole-section averaging, substantially reduced by region-label shuffling, and robust to shifted and rotated partition perturbations. Exploratory ridge and Bayesian analyses highlight a candidate positive fibroblast–ECM association as the most stable bridge-feature relationship in the small CRC–BC design. Supplementary applicability tests in lung, prostate, and ovarian cancer Visium HD sections further show that the same region-aware workflow can be applied across additional solid-tumor contexts. Together, these results support region-aware bridge modeling as a compact and auditable intermediate representation for spatial transcriptomic analysis.

## 2. Methods

### 2.1 Study design and analysis scope

We developed a region-aware bridge modeling framework to derive interpretable mesoscale representations from spatial transcriptomic tissue sections. The analytical objective was to transform spot-level or bin-level biological program information into compact region-level summaries that preserve coarse spatial organization while remaining simple enough for cross-section comparison and exploratory statistical modeling.

The primary proof-of-concept analysis used two public 10x Genomics Visium HD spatial transcriptomic tissue sections: one colorectal cancer (CRC) section and one breast cancer (BC) section. Each section was treated as a spatial field composed of transcriptomic measurements with associated tissue coordinates. Because the primary analysis contained one CRC section and one BC section, all spatial validation was interpreted as within-section validation rather than slide-level, patient-level, or population-level benchmarking. The joint CRC–BC statistical design matrix contained eight region-level observations, corresponding to four median-quadrant regions per section. Ridge and Bayesian analyses were therefore interpreted as regularized exploratory association models and uncertainty-aware sensitivity analyses, not as confirmatory predictive, causal, or population-level models.

To address generalizability without overextending the main analysis, we also performed supplementary external applicability tests using three additional public solid-tumor Visium HD sections: lung cancer, prostate cancer, and ovarian cancer. These external analyses were limited to marker-program bridge scoring, median-quadrant aggregation, within-section heterogeneity analysis, region-label shuffle nulls, and partition-sensitivity analysis. They were not incorporated into the primary CRC–BC ridge or Bayesian models.

### 2.2 Primary CRC and BC Visium HD datasets

The primary datasets were obtained from the 10x Genomics datasets portal at https://www.10xgenomics.com/datasets. The CRC section was from *Visium HD Human Colon Cancer—Gene Expression Library of Colon Cancer (Visium HD) using the Human Whole Transcriptome Probe Set.* The BC section was from *Visium HD Spatial Gene Expression Library, Human Breast Cancer (Fresh Frozen).* These datasets were selected as representative solid-tumor sections with sufficient spatial coverage for region-aware aggregation and within-section validation. For relevant background on high-resolution tumor microenvironment mapping and CRC-focused spatial transcriptomic profiling, see Janesick *et al*. 2023 and Oliveira *et al*. 2025.

Annotated *h5ad* files were used as the starting point for primary bridge-target construction. The CRC object contained 516 363 retained spots/bins and the BC object contained 434 081 retained spots/bins after removal of zero-count observations. No additional rows were removed for missing coordinates or core bridge features during patch-index standardization.

### 2.3 Preprocessing and bridge-target construction

We constructed a transparent spot-level bridge-target representation rather than treating the bridge layer as an unspecified black-box model. For each spatial observation 𝒾, we generated a 21-dimensional bridge-target vector containing seven coarse cell-state indicator dimensions, twelve curated gene-program scores, and two quality-control summaries:


(1) $$\begin{array}{c} z_{𝒾}=(c_{𝒾},\;g_{𝒾},\;q_{𝒾}),\\ c_{𝒾}=(\mathrm{epilike},\;\mathrm{fibroblast},\;\mathrm{endothelial},\;\mathrm{myeloid},\;\mathrm{lymphoid}/\\ \mathrm{plasma},\;\mathrm{smooth}/\mathrm{myoepithelial},\;\mathrm{stressed}),\\ \begin{array}{c} g_{𝒾}=(\mathrm{IFNG},\;\mathrm{IFN}1,\;\mathrm{CYTOTOX},\;\mathrm{AP}/\mathrm{MHC},\;\mathrm{NFKB},\;\\ \mathrm{PROLIF},\;\mathrm{HYPOXIA},\;\mathrm{EMT},\;\mathrm{OXPHOS},\;\mathrm{UPR},\;\mathrm{ECM},\;\mathrm{ANGIO}),\\ q_{𝒾}=(\log(1+\mathrm{total}\;\mathrm{counts}),\;\log(1+\mathrm{detected}\;\mathrm{counts})). \end{array} \end{array}$$

Here, $c_{𝒾}$ denotes the seven coarse cell-state indicator dimensions, $g_{𝒾}$ denotes the twelve curated gene-program scores, and $q_{𝒾}$ denotes the two quality-control summaries.

The cell_type_coarse field used for bridge-target construction was generated during the author’s preprocessing and annotation workflow rather than being taken directly from the source datasets as harmonized coarse annotations. Dataset-specific cell-state labels were harmonized into a common coarse-label scheme to define seven bridge-state indicators. These harmonized annotations were used only to construct the spot-level bridge-target features and were not used as region labels, model outcomes, or downstream statistical targets.

The coarse cell-state indicators were derived from the cell_type_coarse annotation associated with each observation. For CRC, *Epithelial/Tumor* and *Secretory_Epithelial* annotations were mapped to the epithelial-like bridge dimension; *Fibroblast, Endothelial, Myeloid/Inflammatory_Myeloid, Plasma, SmoothMuscle*, and *Stressed/Metabolic* annotations were mapped to the corresponding bridge-state indicators. For BC, *Luminal_Epithelial, Secretory_Epithelial*, and *Basal/Progenitor* annotations were mapped to the epithelial-like bridge dimension; *Fibroblast, Myoepithelial, Immune_Plasma_like*, and *Stressed_Metabolic* annotations were mapped to the corresponding bridge-state indicators. These composition-like dimensions were encoded as binary indicators.

Curated gene-program scores were computed using *scanpy.tl.score_genes*. Programs included IFN-γ response, type-I interferon response, cytotoxicity, antigen presentation/MHC, NF-κB signaling, proliferation, hypoxia, epithelial–mesenchymal transition, oxidative phosphorylation, unfolded-protein response, extracellular matrix, and angiogenesis. If fewer than two genes from a program were present, the corresponding score was set to zero to avoid unstable single-marker scoring. The program gene sets are provided in Supplementary Data 1, available as supplementary material at *Bioinformatics Advances* online.

Expression matrices were inspected before program scoring. If the maximum expression value was less than or equal to 50, the matrix was treated as already normalized or log-transformed; otherwise, counts were normalized using *scanpy.pp.normalize_total* with target sum 10^4^ followed by *scanpy.pp.log1p*. For the primary CRC and BC objects, normalization was skipped because the matrices already appeared log-transformed, with maximum value 9.210 in both datasets. Bridge-target run summaries, feature summaries, and gene-set definitions are provided in Supplementary Data 1, available as supplementary material at *Bioinformatics Advances* online.

### 2.4 Core-four bridge features

The main region-aware analysis focused on four bridge features selected from the 21-dimensional bridge-target representation: epithelial-like, fibroblast, smooth/myoepithelial, and ECM. These features were selected because they capture broad epithelial, stromal, contractile/myoepithelial, and matrix-remodeling axes relevant to solid-tumor spatial organization. The four-feature core bridge vector used in the main region-aware analysis was therefore defined as a pre-specified subset, or projection, of the full 21-dimensional bridge-target vector in Equation (1). For each observation *i*, the core bridge vector was


(2) $$b_{𝒾}=(b_{𝒾,\mathrm{epi}},b_{𝒾,\mathrm{fib}},b_{𝒾,\mathrm{smooth}},b_{𝒾,\mathrm{ECM}})$$

Where $b_{𝒾,k}$ denotes the bridge value for program *k*. These four features were selected a priori because they represent interpretable epithelial–stromal organization, are shared across the CRC and BC sections analyzed in the primary proof-of-concept study, and provide a reduced feature set suitable for the eight-region statistical design. The reduced core-four representation was used to avoid over-parameterizing region-level comparisons while preserving biologically interpretable axes of tissue organization. The four features were not selected on the basis of ridge or Bayesian model performance. These core bridge values served as fixed inputs for spatial aggregation and downstream exploratory modeling. Region labels, dataset labels, ridge outcomes, and Bayesian outcomes were not used to construct the spot-level bridge representation.

### 2.5 Patch-index standardization

Patch-index tables were standardized to provide a reproducible link between bridge features and spatial coordinates. For each primary dataset, the standardized full table retained the 21 bridge dimensions together with barcode, dataset label, sample identifier, coordinate columns, and image-path metadata. A core-four table retaining epithelial-like, fibroblast, smooth/myoepithelial, and ECM bridge features was then generated for the main region-aware analyses. The standardized CRC table contained 516 363 rows and the standardized BC table contained 434 081 rows, with no rows removed for missing coordinates or core bridge features. Column inventories and patch-index summaries are provided in Supplementary Data 1, available as supplementary material at *Bioinformatics Advances* online.

### 2.6 Median-quadrant region-aware aggregation

To capture coarse mesoscale spatial structure, each primary tissue section was partitioned into four median-based quadrants using the original x0 and y0 coordinates. The median x0 and y0 values were used as the partition thresholds, yielding upper-left (Q1_UL), upper-right (Q2_UR), lower-left (Q3_LL), and lower-right (Q4_LR) regions. The fixed 2 × 2 scheme was used as a deliberately simple, reproducible baseline regionalization because harmonized histology-guided regions or pathologist annotations were not available across all public sections. We therefore do not interpret median quadrants as biologically privileged or superior to adaptive spatial clustering, histology-guided domains, graph neighborhoods, or other data-driven regionalizations; instead, we evaluated whether this baseline partition captured non-random regional structure using shuffle-null and shifted/rotated partition-sensitivity analyses.

For each bridge feature *k* and region gamma, the primary region-level summary was the mean bridge value:


(3) $${\bar{b}}_{\gamma ,\kappa }=\frac{1}{n_{\gamma }}\sum_{𝒾\in \gamma }b_{𝒾,\kappa }$$

Where $n_{\gamma }$ is the number of observations assigned to region γ. In addition to the mean, region-level summaries retained the standard deviation, quartiles, minimum, maximum, region size, coordinate bounds, and positive fraction for binary-like bridge features using threshold 0.5. The resulting primary regional summary contained four CRC regions and four BC regions.

### 2.7 Within-section spatial validation

Because the primary CRC and BC analyses each used one tissue section, we designed within-section validation analyses rather than slide-level benchmarking. First, we compared whole-section bridge means with median-quadrant regional summaries. For each dataset and bridge feature, within-section heterogeneity was quantified using regional range, regional standard deviation, coefficient of variation, mean absolute pairwise regional difference, mean absolute deviation from the whole-section mean, and maximum absolute deviation from the whole-section mean. Overall heterogeneity summaries were computed by averaging feature-level metrics across the four core bridge features.

Second, we performed a region-label shuffle null. In each permutation, observed region labels were randomly permuted across observations while preserving the original number of observations per region. This preserved the marginal bridge-feature distribution and region sizes while disrupting spatial localization. For each dataset, feature, and metric, the observed statistic was compared with its shuffle-null distribution. We reported the observed value, null mean, null standard deviation, standardized null *z*-score, empirical upper-tail *P* value, and number of permutations. The final primary validation used 5000 permutations with random seed 0.

Third, we evaluated partition sensitivity using shifted and rotated 2 × 2 schemes. The shifted partition moved the median coordinate thresholds by 10% of the coordinate span, and the rotated partition applied a 45-degree coordinate rotation before quadrant assignment. Regional means and heterogeneity metrics were recomputed under each alternative scheme. Partition sensitivity was summarized by metric changes relative to the original median-quadrant partition and by rank correlations between original and alternative regional mean profiles.

### 2.8 Joint CRC–BC design matrix

After regional aggregation, CRC and BC quadrant summaries were combined into a joint design matrix containing eight observations. The matrix included dataset label, region label, number of observations per region, raw region-level bridge means, and standardized bridge features. For downstream ridge and Bayesian modeling, each bridge feature was standardized across the eight joint regional observations using the population standard deviation:


(4) $${𝓏}_{\gamma ,\kappa }=\frac{{\bar{b}}_{\gamma ,\kappa }-\mu_{\kappa }}{\sigma_{\kappa }},$$

Where $\mu_{\kappa }$ and $\sigma_{\kappa }$ are the mean and population standard deviation of bridge feature *k* across the joint CRC–BC design matrix. The design matrix retained a dataset indicator and three region indicators, with Q1_UL as the reference region. Because this matrix contains only eight observations, all downstream statistical modeling was interpreted as exploratory.

### 2.9 Regularized exploratory ridge modeling

We fit ridge regression models to summarize candidate associations among region-level bridge features. For each bridge target, every candidate model retained the dataset indicator and three region indicators. Candidate bridge panels were formed from all subsets of the remaining non-target bridge features. Ridge penalization was controlled by *α*, evaluated on a log-spaced grid from 10^−3^ to 10^3^. Predictors were standardized within each training fold, and model performance was assessed using leave-one-out cross-validation (LOOCV) across the eight regional observations.

Performance was summarized by RMSE, mean absolute error, cross-validated *R*^2^, and Pearson correlation between observed and predicted values. The selected model for each target minimized LOOCV RMSE, with ties resolved in favor of fewer bridge predictors and then smaller *α*. Final selected coefficients were refit on all eight observations and reported on the standardized-predictor scale. These ridge models were used as regularized exploratory association models, not as confirmatory prediction or causal models.

### 2.10 Bayesian uncertainty-aware sensitivity modeling

Bayesian Gaussian regression models were fit as uncertainty-aware companions to the ridge-selected panels. Each Bayesian model used the same predictor terms selected by ridge modeling. The likelihood was


(5) $${𝓎}_{\gamma }∼N(\eta_{\gamma },\sigma ),$$

$$\eta_{\gamma }=\beta_{0}+\sum 𝒿\beta_{𝒿}{𝓍}_{\gamma ,𝒿},$$

Where ${𝓎}_{\gamma }$ is the standardized region-level bridge target and ${𝓍}_{\gamma ,𝒿}$ are the selected standardized predictors and adjustment covariates.

Models were fit in PyMC using the No-U-Turn Sampler. The final run used four chains, 3000 tuning/warm-up iterations per chain, 2000 posterior draws per chain, and *target_accept *= 0.99. Priors were weakly regularizing: β_0_ ∼ N(0, 1), β_j_ ∼ N(0, 0.5), and σ ∼ HalfNormal (1). Posterior summaries used 94% highest-density intervals. Diagnostics included R-hat, bulk and tail effective sample size, Monte Carlo standard error, and divergent transitions.

Because the design matrix contained eight regional observations, Bayesian results were interpreted as sensitivity analyses. The ECM and smooth/myoepithelial models showed stable sampling diagnostics without divergent transitions. More parameter-rich epithelial-like and fibroblast models retained some divergent transitions despite stronger regularization and high target acceptance; these posterior summaries were therefore interpreted cautiously and were not used as confirmatory evidence.

### 2.11 Supplementary external solid-tumor applicability tests

To evaluate portability of the workflow beyond the two primary sections, we performed supplementary analyses of three additional public solid-tumor Visium HD sections from the 10x Genomics datasets portal: lung cancer, prostate cancer, and ovarian cancer. These external analyses were deliberately limited in scope and were not incorporated into the primary CRC–BC ridge or Bayesian models.

Because harmonized coarse cell-state annotations were not available across all external sections, we used a marker-program version of the core-four bridge representation. The external bridge axes were epithelial-like, fibroblast/stromal, smooth/contractile, and ECM. Marker-program scores were computed using *scanpy.tl.score_genes* after normalization to 10^4^ counts per observation followed by log transformation. Zero-count observations were removed before normalization and scoring. The final retained external observations were 605 431 for lung cancer, 550 444 for prostate cancer, and 450 986 for ovarian cancer.

For each external section, we repeated the same median-quadrant aggregation, heterogeneity metrics, region-label shuffle nulls, and shifted/rotated partition-sensitivity analyses used for the primary sections. The final external validation used 2000 permutations. These analyses were interpreted as supplementary workflow-applicability tests rather than disease-specific biological case studies.

### 2.12 Relationship to existing spatial scale-characterization frameworks

We positioned region-aware bridge modeling relative to existing spatial scale-characterization frameworks, including ATHENA (Martinelli and Rapsomaniki 2022) and MISTy (Tanevski *et al*. 2022), in a supplementary methodological comparison table. ATHENA (Martinelli and Rapsomaniki 2022) is designed for graph-based tumor heterogeneity scoring, whereas MISTy (Tanevski *et al*. 2022) is designed for explainable multi-view modeling of spatial feature relationships. The present framework addresses a narrower and complementary objective: constructing an interpretable bridge-to-region representation from spot-level or bin-level biological program values. We therefore view region-aware bridge modeling as a lightweight mesoscale representation step that can support, rather than replace, graph-based heterogeneity scoring or multi-view spatial-dependency modeling.

### 2.13 Software, outputs, and reproducibility

All analyses were rerun using a cleaned analysis pipeline with reproducible output inventories. Analyses were implemented in Python 3.11.15 with numpy 2.4.3, pandas 2.3.3, scipy 1.17.1, scikit-learn 1.8.0, scanpy 1.11.5, anndata 0.12.11, torch 2.10.0, pymc 5.28.4, and arviz 0.23.4. Random seeds were fixed for permutation tests, ridge model evaluation, and Bayesian sampling where applicable. The supplementary package includes bridge-target definitions, patch-index summaries, regional bridge summaries, validation outputs, ridge and Bayesian model outputs, external solid-tumor applicability outputs, software versions, and a pipeline inventory.

## 3. Results

### 3.1 Bridge-target construction defines interpretable spot-level tissue programs

**Figure 1:**
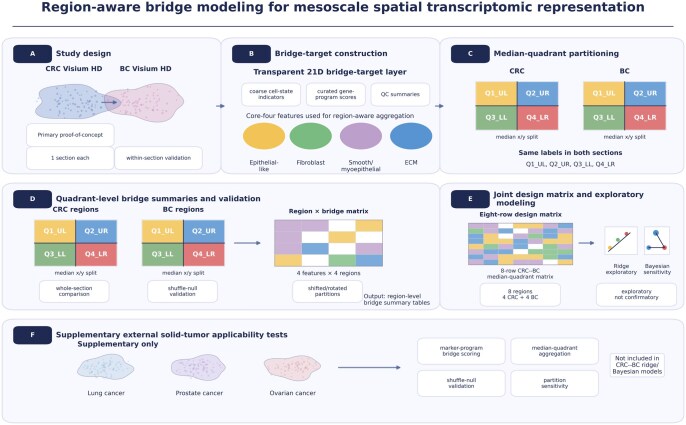
Region-aware bridge modeling workflow for mesoscale spatial transcriptomic representation. Schematic overview of the region-aware bridge modeling workflow. (A) Primary proof-of-concept design using one colorectal cancer (CRC) and one breast cancer (BC) Visium HD spatial transcriptomic section, each analyzed as a within-section spatial field. (B) Bridge-target construction. A transparent 21-dimensional bridge-target layer was constructed from coarse cell-state indicators, curated gene-program scores, and quality-control summaries; the main analysis focused on epithelial-like, fibroblast, smooth/myoepithelial, and extracellular matrix (ECM) features. (C) Median-quadrant partitioning. CRC and BC sections were partitioned using the same median x/y split and four region labels: Q1_UL, Q2_UR, Q3_LL, and Q4_LR. (D) Region-level bridge summaries and validation. Core bridge features were averaged within quadrants and evaluated using whole-section comparisons, region-label shuffle nulls, and shifted/rotated partition-sensitivity analyses. (E) Joint design matrix and exploratory modeling. Four CRC regions and four BC regions were combined into an eight-row design matrix for exploratory ridge modeling and Bayesian sensitivity analysis. (F) Supplementary external solid-tumor applicability tests. Lung, prostate, and ovarian cancer Visium HD sections were analyzed using marker-program bridge scoring, median-quadrant aggregation, shuffle-null validation, and partition-sensitivity analysis.

We first constructed a transparent bridge-target representation for the primary CRC and BC sections (Fig. 1A and B). Rather than treating the bridge layer as an unspecified black-box output, we defined a 21-dimensional spot-level representation composed of coarse cell-state indicators, curated gene-program scores, and quality-control summaries. The main analysis focused on four core bridge features: epithelial-like, fibroblast, smooth/myoepithelial, and ECM. These features were selected to summarize broad epithelial, stromal, contractile/myoepithelial, and matrix-remodeling axes relevant to solid-tumor tissue organization.

The bridge-target construction retained 516 363 observations in the CRC section and 434 081 observations in the BC section. Both matrices were consistent with log-transformed expression values and therefore did not require additional normalization before program scoring. Standardized patch-index tables linked each observation to spatial coordinates and core bridge features, providing the input for region-aware aggregation. This cleaned representation established an auditable bridge-feature layer for all downstream analyses.

### 3.2 Median-quadrant aggregation reveals mesoscale CRC and BC bridge structure

**Figure 2:**
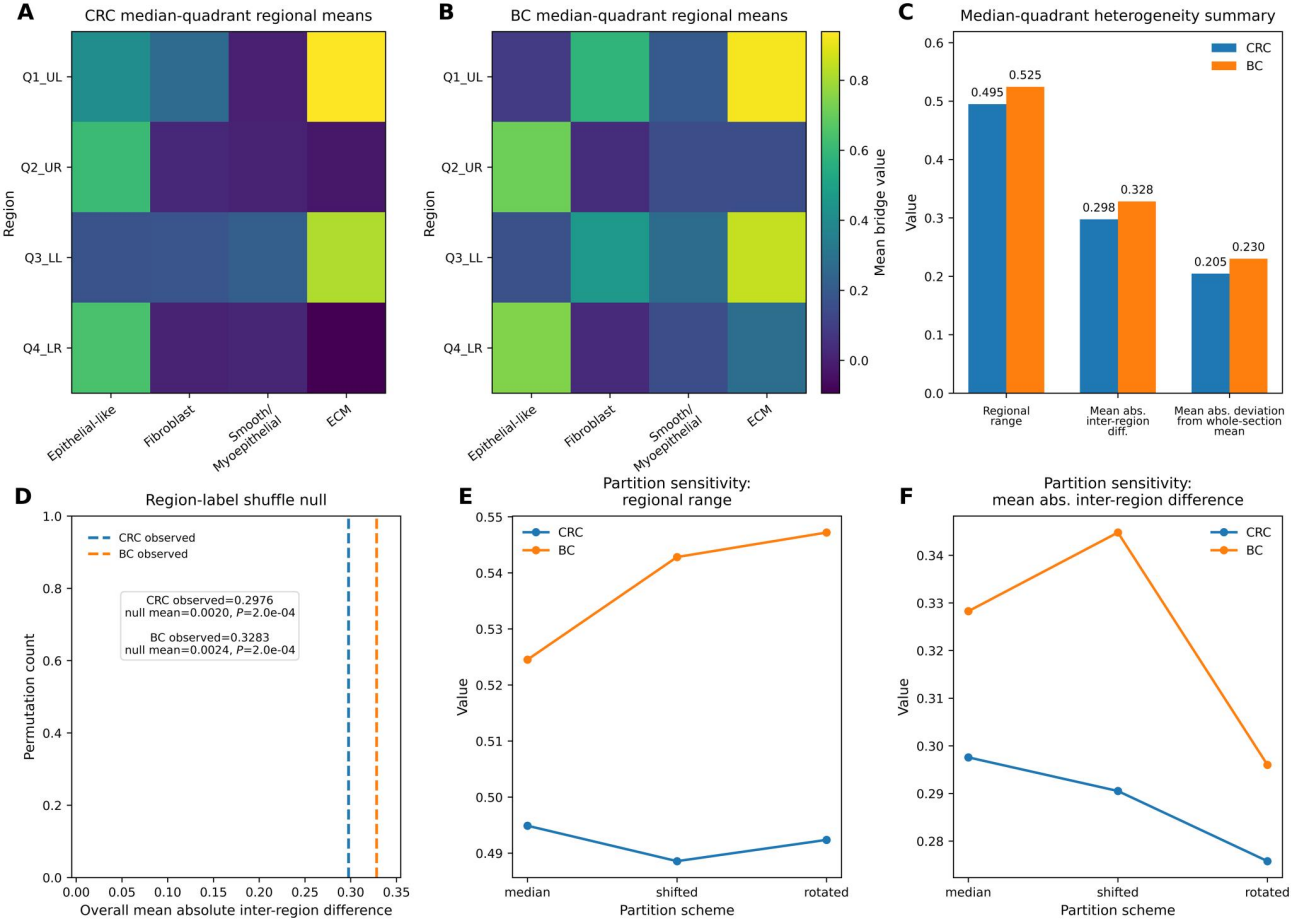
Within-section validation of median-quadrant region-aware bridge summaries. Validation analyses showing that median-quadrant bridge summaries preserve non-random mesoscale spatial heterogeneity in the primary CRC and BC sections. (A, B) Regional mean bridge-feature heatmaps for CRC and BC. Rows indicate median-based quadrants, and columns indicate epithelial-like, fibroblast, smooth/myoepithelial, and ECM bridge features. (C) Heterogeneity summary showing overall regional range, mean absolute inter-region difference, and mean absolute deviation from the whole-section mean. (D) Region-label shuffle-null validation for the overall mean absolute inter-region difference. Null distributions were generated by permuting region labels while preserving region sizes and leaving bridge-feature values unchanged. Observed values were far outside the shuffle-null distributions in both sections, with empirical upper-tail *P *= 2.0 × 10^−4^ based on 5000 permutations. (E, F) Partition-sensitivity analysis comparing the original median-quadrant partition with shifted and rotated 2 × 2 partitions. Similar values across partition perturbations indicate that the observed heterogeneity was not driven by a single exact quadrant boundary.

Each primary tissue section was partitioned into four median-based spatial quadrants, yielding upper-left, upper-right, lower-left, and lower-right regions (Fig. 1C; Fig. S1A, B, available as supplementary material at *Bioinformatics Advances* online). Aggregating the core bridge features within these quadrants produced a compact eight-row CRC–BC design matrix, with four regions per section (Fig. 1D, E). The resulting region-level summaries revealed structured spatial variation rather than uniform section-wide signal. In CRC, epithelial-like values were highest in the upper-right and lower-right quadrants, whereas ECM values were highest in the upper-left and lower-left quadrants. In BC, fibroblast and ECM values were enriched in the upper-left and lower-left quadrants, whereas epithelial-like values were highest in the upper-right and lower-right quadrants (Fig. 2A, B; Fig. S1C, D, available as supplementary material at *Bioinformatics Advances* online).

This compression preserved interpretable mesoscale organization while reducing the data to a small number of region-level observations suitable for validation and exploratory modeling. Because this design matrix contained only eight observations, subsequent statistical models were interpreted conservatively as exploratory summaries.

### 3.3 Within-section validation supports non-random spatial heterogeneity

Because each primary dataset contained one tissue section, we evaluated spatial structure using within-section validation rather than slide-level benchmarking. Median-based 2 × 2 quadrant aggregation revealed substantial mesoscale heterogeneity in both sections. The overall mean absolute pairwise regional difference was 0.298 in CRC and 0.328 in BC, and the mean absolute deviation from the corresponding whole-section mean was 0.205 and 0.230, respectively (Fig. 2C).

Region-label shuffle-null analysis showed that this signal depended on spatial organization. When region labels were randomly permuted while preserving region sizes and the marginal bridge-feature distributions, the overall mean absolute pairwise regional difference decreased from 0.298 to a null mean of 0.0020 in CRC and from 0.328 to a null mean of 0.0024 in BC. Based on 5000 permutations, the empirical upper-tail *P*value was 2.0 × 10^−4^ for both sections (Fig. 2D; Fig. S2A, B, available as supplementary material at *Bioinformatics Advances* online). These results indicate that the observed regional bridge structure is unlikely to reflect feature abundance alone.

Partition-sensitivity analyses further supported robustness to modest changes in regionalization. In BC, the overall mean absolute pairwise regional difference changed from 0.328 under the original median-quadrant partition to 0.345 under the shifted partition (+5.0%) and 0.296 under the rotated partition (–9.8%). In CRC, the corresponding values were 0.298, 0.291 (–2.4%), and 0.276 (–7.3%). Thus, shifted and rotated partitions changed the overall metric by less than 10% in both datasets, supporting that the mesoscale signal was not driven by a single exact quadrant boundary (Fig. 2E, F; Fig. S2C, D, available as supplementary material at *Bioinformatics Advances* online).

### 3.4 Exploratory ridge and Bayesian models summarize candidate bridge-feature associations

**Figure 3:**
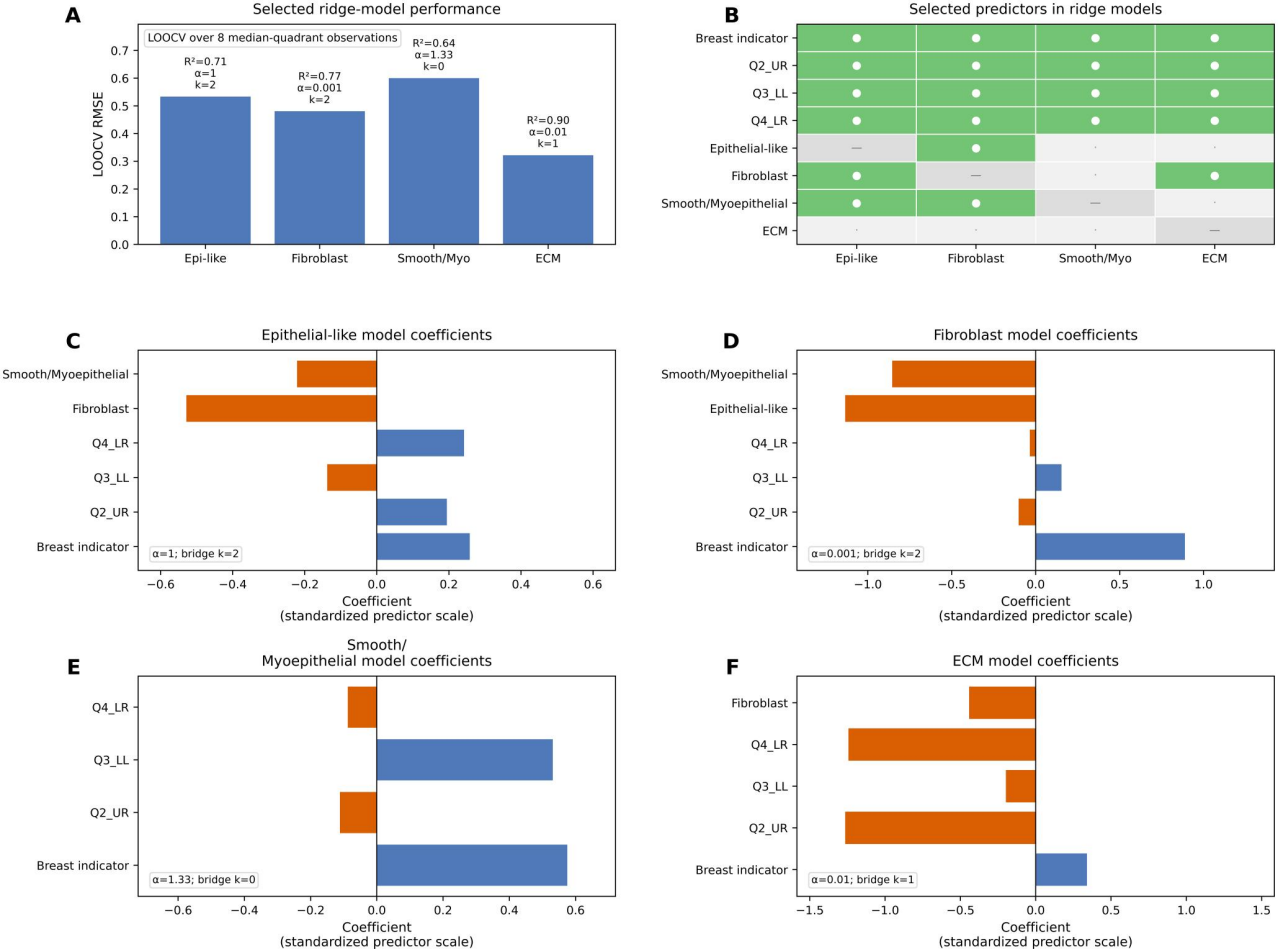
Regularized exploratory ridge modeling of region-level bridge features. Ridge models were used as exploratory association analyses of the eight-row CRC–BC median-quadrant design matrix. (A) Leave-one-out cross-validation (LOOCV) performance for the selected ridge model for each bridge target. Bars show LOOCV RMSE, with cross-validated *R*^2^, selected ridge penalty *α*, and the number of retained non-target bridge predictors (*k*) indicated above each target. (B) Selected predictors in the ridge models. Dataset and region indicators were retained as adjustment terms in all models, while non-target bridge predictors were selected by LOOCV; gray diagonal cells indicate target features that were not eligible to predict themselves. (C–F) Standardized coefficient summaries for selected ridge models for epithelial-like, fibroblast, smooth/myoepithelial, and ECM targets. The ECM model retained fibroblast as the only non-target bridge predictor, whereas smooth/myoepithelial variation was best summarized by dataset and region adjustment terms alone. These models were interpreted as descriptive exploratory associations rather than confirmatory predictive or causal models.

We next used the eight-row CRC–BC median-quadrant design matrix for regularized exploratory association modeling. Each ridge model retained dataset and region indicators, and candidate non-target bridge panels were selected by leave-one-out cross-validation. The selected ECM model retained fibroblast as the only non-target bridge predictor and showed the strongest cross-validated performance among the four exploratory target models (LOOCV *R*^2^ = 0.895, RMSE = 0.324; Fig. 3A and B). Epithelial-like and fibroblast targets selected compact two-feature bridge panels, whereas smooth/myoepithelial variation was best summarized by dataset and region terms alone (Fig. 3C–F). These results support the use of ridge modeling as a descriptive tool for identifying candidate mesoscale bridge-feature relationships while avoiding strong inference from the small eight-region design.

**Figure 4:**
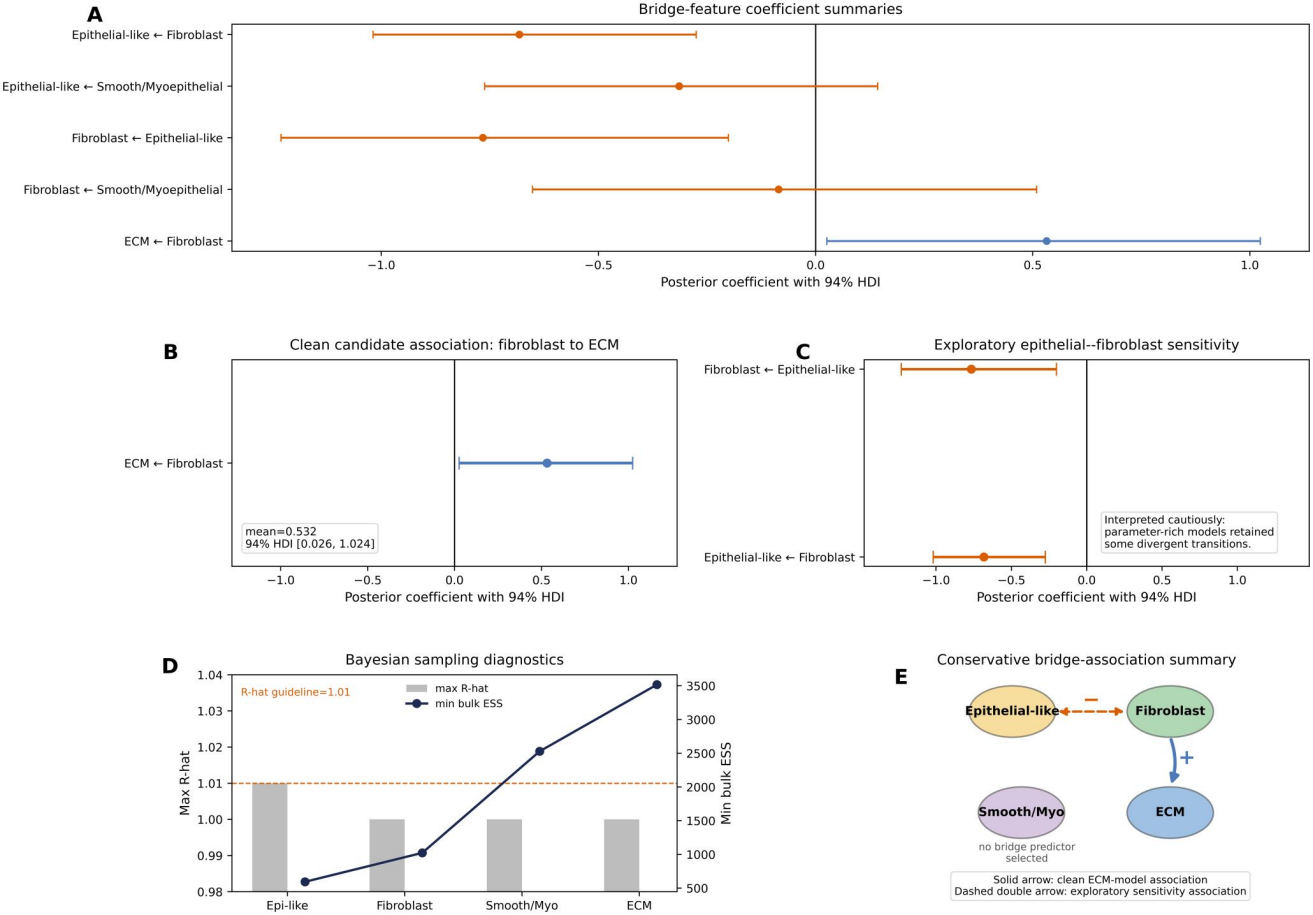
Bayesian sensitivity modeling of region-level bridge-feature associations. Bayesian Gaussian regression models were fit as uncertainty-aware sensitivity analyses corresponding to the ridge-selected panels. (A) Posterior summaries for selected bridge-feature coefficients, shown as posterior means with 94% highest-density intervals (HDIs). (B) Candidate fibroblast–ECM association. The fibroblast coefficient in the ECM target model was positive, with posterior mean 0.532 and 94% HDI 0.026–1.024, and the model showed stable diagnostics without divergent transitions. (C) Exploratory epithelial–fibroblast sensitivity associations. Epithelial-like and fibroblast models showed negative bridge-feature coefficients, but these more parameter-rich models retained some divergent transitions and were interpreted cautiously. (D) Bayesian sampling diagnostics. Bars show maximum by target model, and the line shows minimum bulk effective sample size; the dashed horizontal line indicates the 1.01 guideline. (E) Conservative conceptual bridge-association summary. The solid arrow denotes the cleaner ECM-model fibroblast association, whereas the dashed double arrow denotes exploratory epithelial–fibroblast sensitivity associations.

Bayesian Gaussian regression models were then fit as uncertainty-aware companions to the ridge-selected panels. Diagnostic behavior varied with model complexity. The ECM and smooth/myoepithelial models showed stable sampling diagnostics without divergent transitions. In the ECM model, the fibroblast bridge coefficient was positive, with posterior mean 0.532 and 94% HDI 0.026–1.024, supporting a candidate positive mesoscale association between fibroblast-like and ECM bridge structure (Fig. 4A and B). Models for epithelial-like and fibroblast targets had acceptable and effective sample sizes after stronger shrinkage but retained some divergent transitions; these posterior summaries were therefore treated as exploratory sensitivity results rather than definitive estimates (Fig. 4C–E).

### 3.5 Supplementary external solid-tumor sections support workflow applicability

To evaluate whether the workflow was applicable beyond the two primary CRC and BC sections, we performed supplementary analyses of three additional public solid-tumor Visium HD sections: lung cancer, prostate cancer, and ovarian cancer (Fig. 1F). These analyses were intentionally restricted to marker-program bridge scoring, median-quadrant aggregation, within-section heterogeneity, region-label shuffle nulls, and partition-sensitivity analysis. They were not incorporated into the primary CRC–BC ridge or Bayesian models.

Across all three external sections, observed overall regional heterogeneity exceeded the shuffle-null expectation based on 2000 permutations. The observed overall mean absolute pairwise regional difference was 0.0259 in lung cancer compared with a null mean of 0.00077, 0.0648 in prostate cancer compared with 0.00149, and 0.4008 in ovarian cancer compared with 0.00296. The empirical upper-tail *P* value was 5.0 × 10^−4^ for all three external sections (Supplementary Figs. S3–S4, available as supplementary material at *Bioinformatics Advances* online). These supplementary results support portability of the region-aware aggregation workflow across additional solid-tumor contexts while keeping the primary statistical modeling focused on the CRC–BC proof-of-concept design.

## 4. Discussion

We present a region-aware bridge modeling framework for constructing interpretable mesoscale representations of spatial transcriptomic tissue sections. The central contribution is a reproducible bridge-to-region workflow that transforms spot-level or bin-level biological program values into compact regional summaries. In the primary CRC and BC sections, median-quadrant aggregation preserved spatially organized bridge-feature heterogeneity that was obscured by whole-section averaging, substantially reduced under region-label shuffling, and remained stable under shifted and rotated partitions. These results support region-aware bridge features as a practical intermediate representation between high-resolution spot-level maps and overly compressed whole-section summaries.

An important feature of the framework is that the bridge layer is explicitly defined rather than treated as an unspecified black-box output. The workflow defines bridge-target construction using coarse cell-state indicators, curated gene-program scores, and quality-control summaries. The main analysis focuses on four interpretable axes: epithelial-like, fibroblast, smooth/myoepithelial, and ECM. This transparency is important because region-aware summaries are only as interpretable as the features being aggregated. By providing gene sets, run summaries, patch-index tables, and region-level outputs, the analysis makes the bridge layer auditable and reproducible.

The validation analyses strengthen the rationale for the median-quadrant representation while clarifying its intended scope. We do not claim that four quadrants are universally optimal or that they replace histology-guided domains, graph neighborhoods, or adaptive spatial clustering. Rather, median quadrants provide a minimal and reproducible mesoscale baseline suitable for a small proof-of-concept study. Harmonized pathologist-annotated histological regions were not available across the public Visium HD sections analyzed here, so we could not formally test concordance between quadrant boundaries and pathologist-defined tissue compartments. The observed heterogeneity was far larger than shuffle-null expectations in both primary sections and remained within 10% under shifted and rotated partitions. Thus, the main conclusion is not that the precise quadrant boundaries are biologically privileged, but that region-aware aggregation can capture non-random spatial structure that whole-section averages obscure.

The statistical modeling results should also be interpreted conservatively. The joint CRC–BC design matrix contains eight regional observations, so ridge and Bayesian analyses cannot establish population-level dependency structure. Instead, they provide a compact exploratory summary of candidate bridge-feature associations. Within this scope, the ECM model was the most stable and interpretable: ridge modeling selected fibroblast as the only non-target bridge predictor, and Bayesian sensitivity analysis supported a positive fibroblast–ECM association with stable diagnostics and no divergent transitions. More complex epithelial-like and fibroblast Bayesian models retained some divergences despite stronger regularization and high target acceptance, reinforcing the need for cautious interpretation and larger multi-section datasets.

The supplementary external solid-tumor analyses address workflow portability without expanding the manuscript into a full benchmarking study. Applying the same lightweight workflow to lung, prostate, and ovarian cancer Visium HD sections showed that median-quadrant aggregation and shuffle-null validation could be performed across additional solid-tumor contexts. These analyses were intentionally not added to the primary ridge or Bayesian models. Instead, they demonstrate applicability of the workflow beyond the original CRC and BC sections and provide a foundation for future multi-section evaluation.

Region-aware bridge modeling is also complementary to existing spatial scale-characterization frameworks. ATHENA is designed for graph-based quantification of tumor heterogeneity, whereas MISTy is designed for explainable multi-view modeling of spatial feature relationships. The present framework addresses a narrower and complementary objective: constructing an interpretable bridge-to-region representation from spot-level biological program values. Thus, region-aware bridge modeling should be viewed as a lightweight mesoscale representation step that can support, rather than replace, graph-based heterogeneity scoring or multi-view spatial-dependency modeling.

Several limitations remain. First, the primary statistical analysis is based on one CRC and one BC section, each summarized into four regions. The validation is therefore within-section rather than slide-level, patient-level, or population-level. Second, the median-quadrant partition is intentionally simple and may not align with all histologic or molecular boundaries; harmonized pathologist annotations were not available for formal comparison in this study. Third, the primary bridge features capture a limited set of epithelial, stromal, contractile, and matrix-associated programs; additional immune, vascular, hypoxic, or tumor-subtype-specific programs may be needed in future applications. Fourth, although the external solid-tumor tests support workflow portability, they are supplementary applicability analyses rather than full disease-specific validation studies, and they do not establish generalizability to non-tumor tissues, developmental tissues, inflammatory disease settings, or non-Visium platforms. Finally, the Bayesian models highlight the challenges of uncertainty quantification in very small region-level designs.

Future work should extend this framework to larger multi-section, multi-patient, and multi-platform datasets. Such studies could evaluate adaptive or histology-guided regionalization, compare bridge-to-region summaries with graph-based and multi-view spatial models, and test whether regional bridge states are reproducible across patients or associated with clinical phenotypes. Longitudinal or treatment-matched spatial datasets would further enable evaluation of whether regional bridge states can serve as dynamic variables for monitoring tissue reorganization over time.

In summary, region-aware bridge modeling provides a compact and interpretable mesoscale representation for spatial transcriptomic tissue sections. In primary CRC and BC sections, median-quadrant bridge summaries preserved non-random spatial heterogeneity and supported conservative exploratory modeling. Supplementary analyses in lung, prostate, and ovarian cancer further supported workflow applicability across additional solid-tumor contexts. These findings motivate larger studies that use region-aware bridge features as reproducible intermediate variables for spatial transcriptomic analysis.

## Supplementary Material

vbag176_Supplementary_Data

## Data Availability

The primary datasets were obtained from the 10x Genomics datasets portal. Supplementary external applicability analyses used additional public 10x Genomics Visium HD sections from lung cancer, prostate cancer, and ovarian cancer. Processed outputs generated for this study, including bridge-target summaries, patch-index tables, region-level summaries, validation outputs, ridge and Bayesian model outputs, external applicability outputs, and pipeline inventories, are provided as Supplementary Data. The code used for bridge-target construction, patch-index standardization, median-quadrant aggregation, within-section validation, exploratory ridge modeling, Bayesian sensitivity modeling, and supplementary external solid-tumor applicability tests is available from the project GitHub repository at https://github.com/shkim9391/Region_Aware_Bridge_Modeling. A DOI-backed archived release is available from Zenodo under 10.5281/zenodo.19801620.
