# Supplementary material for: Region-aware bridge modeling enables interpretable mesoscale representation of spatial transcriptomic tissue sections
Source: Bioinform Adv. 2026 Jun 23;6(1):vbag176. doi: 10.1093/bioadv/vbag176 (PMC13317975; doi:10.1093/bioadv/vbag176)
Supplement: vbag176_Supplementary_Data [file vbag176_supplementary_data.zip › Revised_Region_Aware_Bridge_SI.pdf]

Supplementary Information

Region-aware bridge modeling enables interpretable mesoscale representation of spatial transcriptomic tissue sections

Seung-Hwan Kim[1,2,*]
[1]Department of Biology, Fisher College, Boston, MA, USA
[2]Department of Pediatric Oncology, Dana-Farber Cancer Institute, Boston, MA, USA
[*]To whom correspondence should be addressed: `seung-hwan.kim@fisher.edu`

Supplementary data are organized as six logical packages corresponding to the cleaned region-aware bridge-modeling workflow. This organization separates bridge-target construction, primary CRC–BC regional summaries, within-section validation, exploratory modeling, external applicability testing, and reproducibility materials.

Supplementary Methods

Scope of the supplementary analyses

The primary manuscript analyzes one colorectal cancer (CRC) and one breast cancer (BC) Visium HD tissue section as a proof-of-concept for region-aware bridge modeling. Because each primary dataset contains one tissue section, the validation analyses are interpreted as *within-section* spatial validation rather than slide-level, patient-level, or population-level benchmarking. The joint CRC–BC statistical design matrix contains eight region-level observations, corresponding to four median-quadrant regions per section. Ridge and Bayesian analyses are therefore interpreted as exploratory association and sensitivity analyses.

Supplementary external solid-tumor analyses were added to evaluate workflow portability across additional tissue contexts. These external analyses include lung cancer, prostate cancer, and ovarian cancer Visium HD sections. They are intentionally limited to marker-program bridge scoring, median-quadrant aggregation, within-section heterogeneity, region-label shuffle-null validation, and shifted/rotated partition sensitivity. They are not incorporated into the primary CRC–BC ridge or Bayesian models.

Bridge-target construction

The primary bridge-target representation was constructed as a transparent 21-dimensional spot-level feature layer. It included seven coarse cell-state indicator dimensions, twelve curated gene-program scores, and two quality-control summaries. The main manuscript focuses on four core bridge features selected from this representation: epithelial-like, fibroblast, smooth/myoepithelial, and extracellular matrix (ECM). These features were selected because they capture broad epithelial, stromal, contractile/myoepithelial, and matrix-remodeling axes relevant to solid-tumor tissue organization.

The curated gene-program scores were computed using `scanpy.tl.score_genes`. If fewer than two genes from a program were present in a dataset, the corresponding score was set to zero to avoid unstable single-marker scoring. Gene sets used for bridge-target construction and external applicability testing are provided in Supplementary Data 1 and Supplementary Data 5, respectively.

Quality control, normalization, and feature scoring details

Quality control was intentionally limited to removal of zero-count observations and exclusion of observations lacking required spatial coordinates or core bridge features, because the public annotated Visium HD objects had already undergone upstream dataset-specific processing. No additional gene-level filtering, batch correction, or cross-section integration was applied before bridge scoring in the primary CRC–BC analysis.

Before gene-program scoring, expression matrices were inspected to determine whether additional normalization was required. If the maximum expression value was less than or equal to 50, the matrix was treated as already normalized or log-transformed. Otherwise, counts were normalized using `scanpy.pp.normalize_total` with target sum 10^4 followed by `scanpy.pp.log1p`. For the primary CRC and BC objects, normalization was skipped because the matrices appeared already log-transformed. For the supplementary external lung, prostate, and ovarian cancer sections, zero-count observations were removed before normalization, and expression matrices were normalized to 10^4 counts per observation followed by log transformation before marker-program scoring.

Median-quadrant region-aware aggregation

Each primary tissue section was partitioned into four median-based spatial quadrants using the original $x0$ and $y0$ coordinates. The median $x0$ and $y0$ values were used as thresholds, yielding upper-left (`Q1_UL`), upper-right (`Q2_UR`), lower-left (`Q3_LL`), and lower-right (`Q4_LR`) regions. Median-based splitting was used as a simple, reproducible, low-degree-of-freedom regionalization that reduces region-size imbalance relative to a min–max midpoint split. The same labels were used for CRC and BC sections.

For each region r and bridge feature k, the region-level mean was computed as

$$\bar{b}_{r,k} = \frac{1}{n_r} \sum_{i \in r} b_{i,k},$$

where $b_{i,k}$ is the bridge value for observation i and feature k, and n_r is the number of observations in region r. Region-level tables also retain region size, coordinate bounds, dispersion summaries, quantiles, and positive-fraction summaries where applicable.

Rationale and limitations of the fixed 2×2 regionalization

The fixed 2×2 median-quadrant scheme was used as a deliberately simple and reproducible baseline regionalization. This choice was appropriate for a proof-of-concept study because the public sections analyzed here did not provide harmonized pathologist-annotated histological regions across all datasets. Median-based splitting also reduces region-size imbalance relative to min–max midpoint splitting and allows the same regionalization rule to be applied uniformly across CRC, BC, and the supplementary external solid-tumor sections.

We do not interpret the median quadrants as biologically privileged, histology-matching, or superior to adaptive spatial clustering, histology-guided domains, graph neighborhoods, tissue-compartment annotations, or other data-driven regionalization strategies. Rather, the purpose of the fixed 2×2 scheme was to define a low-complexity mesoscale unit that could be inspected directly and stress-tested using region-label shuffle nulls and shifted/rotated partition-sensitivity analyses. In datasets with pathologist-annotated compartments or robust spatial clusters, the same bridge-to-region aggregation procedure could be applied to those alternative region definitions.

Histology annotation availability

Pathologist-annotated histological region labels were not available in a harmonized form for the public Visium HD sections analyzed here. Therefore, we could not formally test concordance between the fixed 2×2 quadrants and pathologist-defined histological compartments. We instead treated median quadrants as a reproducible geometric baseline and evaluated whether the resulting regional summaries captured non-random spatial structure relative to shuffle-null and perturbed-partition controls.

Within-section validation and partition sensitivity

Within-section validation compared median-quadrant summaries against whole-section means, region-label shuffle nulls, and shifted/rotated partitions. Heterogeneity metrics included regional range, regional standard deviation, coefficient of variation, mean absolute pairwise regional difference, mean absolute deviation from the whole-section mean, and maximum absolute deviation from the whole-section mean.

For the shuffle-null analysis, observed region labels were randomly permuted across observations while preserving the original number of observations per region. This preserves the marginal bridge-feature distribution and region sizes while disrupting spatial localization. The final primary validation used 5000 permutations. Partition-sensitivity analyses compared the original median-quadrant scheme with a shifted partition, in which median thresholds were shifted by 10% of the coordinate span, and a rotated partition, in which coordinates were rotated by 45 degrees before assigning quadrants.

Definition of within-section heterogeneity metrics

For each dataset, bridge feature, and regionalization scheme, within-section heterogeneity was summarized using multiple complementary metrics. The regional range was defined as the maximum region-level mean minus the minimum region-level mean across the four regions. The regional standard deviation was computed across the four region-level means. The coefficient of variation was computed as the regional standard deviation divided by the absolute value of the corresponding whole-section mean when the denominator was nonzero.

The mean absolute pairwise regional difference was computed as the average absolute difference across all unordered pairs of region-level means. The mean absolute deviation from the whole-section mean was computed as the average absolute difference between each region-level mean and the corresponding whole-section mean. The maximum absolute deviation from the whole-section mean was the largest such deviation across regions. Overall heterogeneity summaries were computed by averaging feature-level metrics across the four core bridge features.

Ridge and Bayesian modeling details

The primary CRC–BC design matrix contained eight region-level observations, corresponding to four median-quadrant regions from each tissue section. Ridge regression models were used only as regularized exploratory association models. For each bridge target, all candidate models included dataset and region indicators as adjustment terms. Candidate non-target bridge panels were formed from all subsets of the remaining bridge features. Ridge penalties were evaluated over a log-spaced α grid from 10^{-3} to 10^{3}. Predictors were standardized within each leave-one-out cross-validation training fold, and the selected model minimized LOOCV RMSE, with ties resolved in favor of fewer bridge predictors and then smaller α.

Bayesian Gaussian regression models were fit as uncertainty-aware sensitivity analyses corresponding to the ridge-selected panels. Models were fit in `PyMC` using the No-U-Turn Sampler. The final run used four chains, 3000 tuning/warm-up iterations per chain, 2000 posterior draws per chain, and `target_accept`=0.99. Priors were weakly regularizing, with $\beta_0 \sim \mathcal{N}(0, 1)$, $\beta_j \sim \mathcal{N}(0, 0.5)$, and $\sigma \sim \text{HalfNormal}(1)$. Posterior summaries used 94% highest-density intervals. Diagnostics included $\hat{R}$, bulk effective sample size, tail effective sample size, Monte Carlo standard error, and divergent transitions.

Because the design matrix contained only eight observations, these models were not interpreted as confirmatory prediction models, causal models, or population-level dependency estimates. They were used to summarize candidate bridge-feature associations and to evaluate whether selected associations remained stable under regularized and uncertainty-aware modeling.

External solid-tumor applicability tests

For external lung, prostate, and ovarian cancer Visium HD sections, harmonized coarse cell-state annotations were not assumed. Therefore, a marker-program version of the core-four bridge axes

was used: epithelial-like, fibroblast/stromal, smooth/contractile, and ECM. Expression matrices were normalized to a target sum of 10^4 per observation and log-transformed before gene-program scoring. Zero-count observations were removed before normalization and scoring. The retained external observations were 605,431 for lung cancer, 550,444 for prostate cancer, and 450,986 for ovarian cancer.

The same median-quadrant aggregation, heterogeneity summaries, region-label shuffle nulls, and shifted/rotated partition-sensitivity analyses were applied to each external section. The final external validation used 2000 permutations. These analyses are supplementary applicability tests and are not used for the primary ridge or Bayesian statistical models.

Scope of external applicability

The supplementary external analyses extend the workflow from the primary CRC and BC sections to three additional solid-tumor Visium HD sections: lung cancer, prostate cancer, and ovarian cancer. These tests were designed to evaluate whether the bridge-scoring, median-quadrant aggregation, shuffle-null validation, and partition-sensitivity workflow could be applied across additional solid-tumor contexts.

These analyses do not establish applicability to non-tumor tissues, developmental tissues, inflammatory disease settings, or non-Visium spatial transcriptomic platforms. Such evaluations would require additional datasets, harmonized preprocessing, and potentially tissue-specific bridge-program definitions. We therefore interpret the external analyses as workflow-applicability tests rather than full disease-specific validation studies.

Relationship to existing spatial scale-characterization frameworks

Supplementary Table S1 positions region-aware bridge modeling relative to existing spatial scale-characterization frameworks, including ATHENA and MISTy. This comparison is intended to clarify methodological scope rather than to claim direct benchmarking superiority. A full benchmark against graph-based heterogeneity scoring or multiview spatial-dependency modeling would require a separate study design and is beyond the scope of the present proof-of-concept study.

Table S1: Relationship of region-aware bridge modeling to existing spatial scale-characterization frameworks. The comparison clarifies methodological scope and intended use rather than claiming direct benchmarking superiority.

Framework	Primary analytical scale	Main input representation	Primary output	Relationship to this study
ATHENA	Tumor-ecosystem heterogeneity represented through spatial graphs	Spatial omics measurements from cells, spots, or regions	Graph-based heterogeneity and complexity scores for spatial tumor ecosystems	Complementary. ATHENA is designed to quantify spatial tumor heterogeneity, whereas region-aware bridge modeling constructs low-dimensional mesoscale bridge summaries for interpretable regional comparison.
MISTy	Multiple spatial or functional views, including local and neighborhood contexts	Spatially resolved molecular or cellular features	Explainable multiview models of feature relationships across spatial contexts	Complementary. MISTy models spatial dependencies and feature relationships across views, whereas the present framework emphasizes a simple bridge-to-region representation and partition-sensitivity analysis.
Region-aware bridge modeling	Whole section to mesoscale regions, here median-based quadrants	Spot-level or bin-level bridge-program features	Region-level bridge summaries, within-section heterogeneity metrics, shuffle-null validation, and exploratory association models	Designed as a lightweight, reproducible intermediate representation that can be inspected directly and used as input for subsequent spatial modeling.

Reproducibility materials

The supplementary data package provides bridge-target construction summaries, gene-program definitions, patch-index summaries, median-quadrant regional summaries, within-section validation outputs, partition-sensitivity outputs, exploratory ridge and Bayesian model outputs, supplementary external solid-tumor applicability outputs, software versions, and a cleaned pipeline inventory. These files are provided to make the revised workflow auditable and reproducible while keeping the main manuscript focused on the conceptual and analytical results.

Supplementary Figures

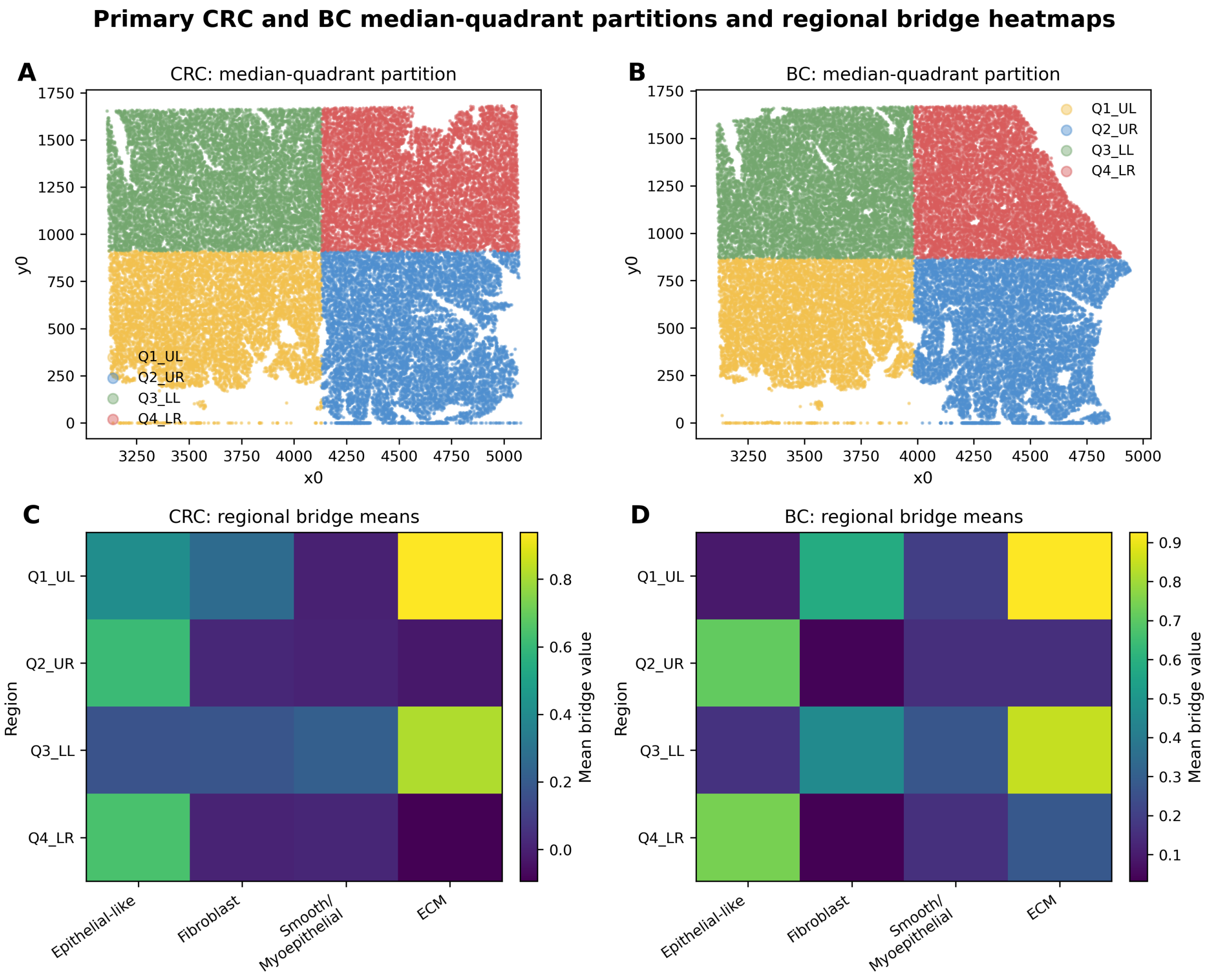

Figure S1: Primary CRC and BC median-quadrant partitions and regional bridge heatmaps. (A,B) Sampled spatial observations from the primary CRC and BC Visium HD sections colored by median-quadrant assignment. Both sections were partitioned using the same median x/y split and the same region labels: `Q1_UL`, `Q2_UR`, `Q3_LL`, and `Q4_LR`. **(C,D)** Region-level heatmaps showing mean bridge-feature values for epithelial-like, fibroblast, smooth/myoepithelial, and ECM features across the four median-quadrant regions in CRC and BC.

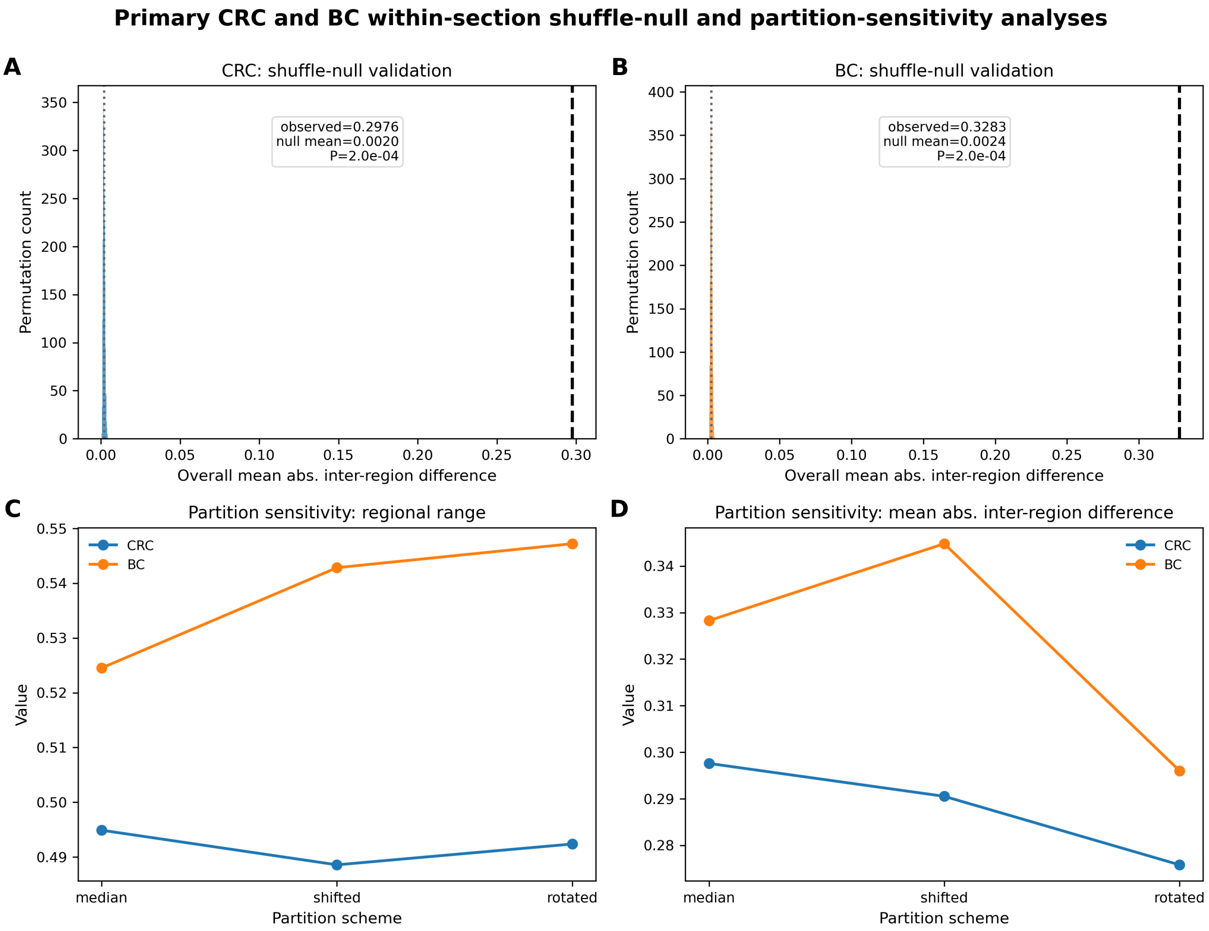

Figure S2: Primary CRC and BC within-section shuffle-null and partition-sensitivity analyses. (A,B) Region-label shuffle-null distributions for the overall mean absolute inter-region difference in the primary CRC and BC sections. Dashed vertical lines show observed values, and dotted vertical lines show shuffle-null means. **(C,D)** Partition-sensitivity analyses comparing the original median-quadrant partition with shifted and rotated 2×2 partitions for overall regional range and mean absolute inter-region difference. These analyses support that the observed regional heterogeneity was not driven by a single exact quadrant boundary.

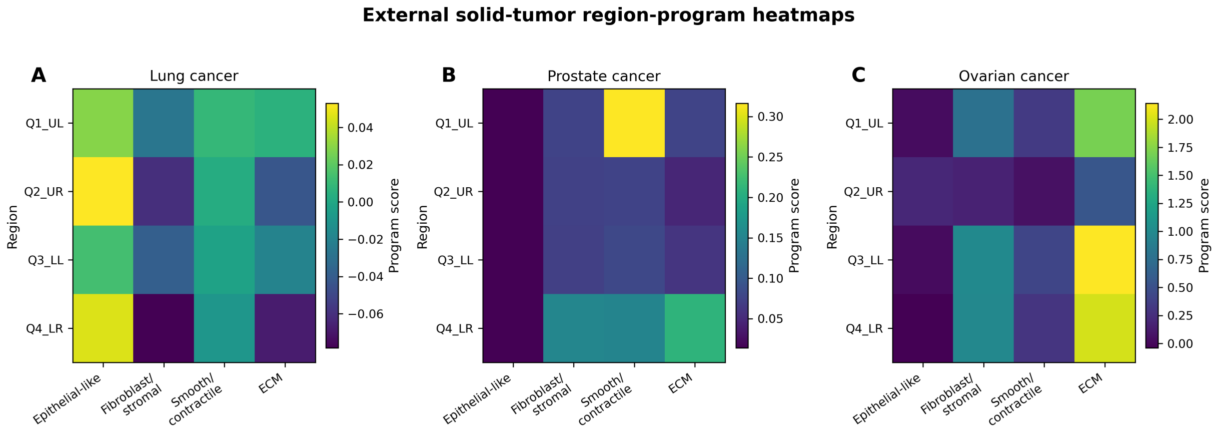

Figure S3: External solid-tumor region-program heatmaps for lung, prostate, and ovarian cancer. Median-quadrant region-program heatmaps for supplementary external solid-tumor applicability tests. **(A)** Lung cancer, **(B)** prostate cancer, and **(C)** ovarian cancer Visium HD sections were analyzed using marker-program bridge axes representing epithelial-like, fibroblast/stromal, smooth/contractile, and ECM programs. These analyses were used only to evaluate workflow portability and were not incorporated into the primary CRC–BC ridge or Bayesian models.

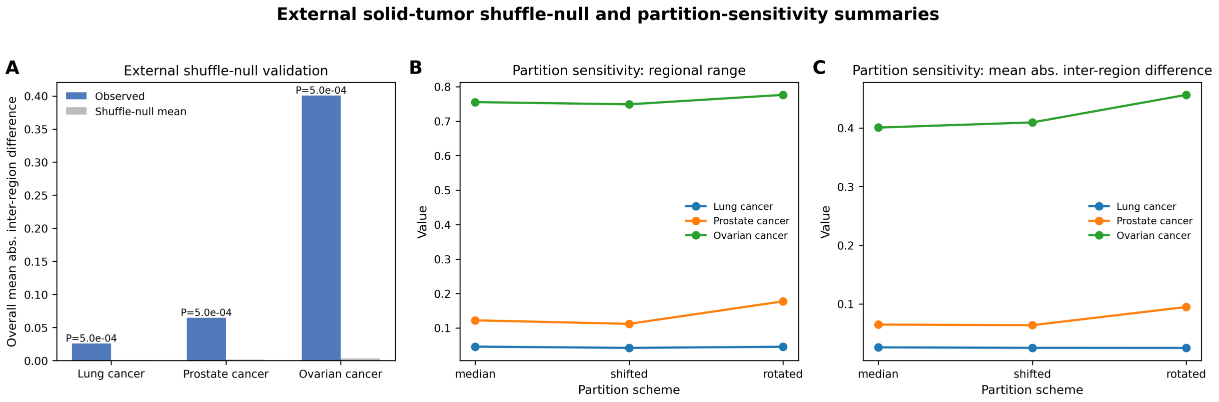

Figure S4: External solid-tumor shuffle-null and partition-sensitivity summaries. Supplementary validation summaries for external lung, prostate, and ovarian cancer Visium HD sections. **(A)** Observed overall mean absolute inter-region differences were compared with shuffle-null means from 2000 region-label permutations. **(B,C)** Partition-sensitivity analyses comparing the original median-quadrant partition with shifted and rotated 2×2 partitions for overall regional range and mean absolute inter-region difference. These supplementary analyses support portability of the region-aware workflow across additional solid-tumor sections.

Supplementary Data

Table S2: Supplementary data organization for the region-aware bridge-modeling workflow.

Table S2: Supplementary data organization for the cleaned region-aware bridge-modeling revision.

Data	Key files	Description	Supports
Supplementary Data 1	bridge_target_gene_sets.csv; bridge_target_summary.csv; bridge_target_run_summary.csv; patch_index_columns.csv; patch_index_summary.csv	Bridge-target construction outputs and gene-program definitions. Includes the 21-dimensional bridge-target representation, core-four bridge-feature definitions, gene sets, preprocessing/normalization status, retained observation counts, and patch-index column inventory.	Methods; bridge-feature construction
Supplementary Data 2	crc_region_bridge_summary_core4_regions4.csv; breast_region_bridge_summary_core4_regions4.csv; combined_region_bridge_summary_crc_breast_core4_regions4.csv; region_bridge_standardization_stats_crc_breast_core4.csv; dataset_region_bridge_summary_crc_breast_core4.csv	Primary CRC–BC region-aware bridge summaries. Includes median-quadrant region-level means, dispersion summaries, quantiles, positive fractions, region sizes, coordinate bounds, and joint standardization statistics for the four core bridge features.	Figs. 1–2; Supplementary Fig. S1
Supplementary Data 3	within_section_whole_means.csv; within_section_region_means_by_scheme.csv; within_section_heterogeneity_metrics_by_scheme.csv; within_section_shuffle_null_summary.csv; within_section_partition_metric_changes.csv; within_section_partition_region_correlations.csv	Within-section validation and partition-sensitivity outputs for the primary CRC and BC sections. Includes whole-section means, regional means under original/shifted/rotated schemes, heterogeneity metrics, shuffle-null summary statistics, and partition-sensitivity summaries.	Fig. 2; Supplementary Fig. S2
Supplementary Data 4	joint_region_bridge_model_matrix_crc_breast_core4.csv; ridge_model_selection_summary.csv; ridge_coefficients_selected_models.csv; ridge_loocv_predictions_selected_models.csv; bayesian_posterior_summary.csv; bayesian_diagnostics.csv; bayesian_model_config.csv	Exploratory ridge and Bayesian sensitivity modeling outputs. Includes the eight-row modeling matrix, ridge model-selection summaries, selected ridge coefficients, LOOCV predictions, Bayesian posterior summaries, diagnostic summaries, and Bayesian sampling configuration.	Figs. 3–4

Data	Representative files	Description	Supports
Supplementary Data 5	`external_program_gene_sets.csv`; `external_program_score_run_summary.csv`; `external_region_program_means_by_scheme.csv`; `external_shuffle_null_summary.csv`; `external_partition_metric_changes.csv`; `external_metrics_summary_combined.csv`	Supplementary external solid-tumor applicability outputs for lung, prostate, and ovarian cancer Visium HD sections. Includes marker-program gene sets, retained observation counts, regional program means, shuffle-null summaries, partition-sensitivity summaries, and 10x metrics summaries.	Supplementary Figs. S3–S4; external applicability tests
Supplementary Data 6	`revision_pipeline_summary.xlsx`; `revision_file_inventory.csv`; `software_versions.txt`	Analysis pipeline summary and reproducibility inventory. Includes the cleaned analysis-step summary, expected output-file inventory, and software/package versions used in the revised analysis.	Reproducibility; pipeline audit trail
